# Educational attainment predicts negative perceptions women have of their own climate change knowledge

**DOI:** 10.1371/journal.pone.0210149

**Published:** 2019-01-04

**Authors:** Kathryn R. Selm, M. Nils Peterson, George R. Hess, Scott M. Beck, Melissa R. McHale

**Affiliations:** 1 Natural Resources Program, Department of Forestry & Environmental Resources, North Carolina State University, Raleigh, North Carolina, United States of America; 2 Fisheries, Wildlife, and Conservation Biology Program, Forestry and Environmental Resources, North Carolina State University, Raleigh, NC, United States of America; 3 Department of Forestry & Environmental Resources, North Carolina State University, Raleigh, North Carolina, United States of America; 4 Natural Resources Ecology Lab, Department of Ecosystem Science & Sustainability, Colorado State University, Fort Collins, CO, United States of America; University of Essex, UNITED KINGDOM

## Abstract

Education may encourage personal and collective responses to climate change, but climate education has proven surprisingly difficult and complex. Self-perception of knowledge and intelligence represent one factor that may impact willingness to learn about climate change. We explored this possibility with a case study in Raleigh, North Carolina in 2015 (n = 200). Our goal was to test how gender and ethnicity influenced perceptions people had of their own climate change knowledge. Survey respondents were asked how strongly they agreed with the statement “I feel knowledgeable about climate change” (1 = strongly disagree, and 5 = strongly agree). Our survey instrument also included demographic questions about race, age, income, gender, and education, as well as respondent’s experience with natural disasters and drought. We observed an interaction between education and gender where women’s self-perceived knowledge was higher than men among people with low levels of educational attainment, but was higher for men than women among people with high levels of educational attainment. In addition, minority respondents self-reported lower perceived climate change knowledge than white respondents, regardless of educational attainment. This study enhances our understanding of the gender gap in self-perceptions of climate knowledge by suggesting it is contingent on educational attainment. This could be the result of stereotype-threat experienced by women and minorities, and exacerbated by educational systems. Because people who question their knowledge are often more able to learn, particularly in ideologically charged contexts, highly educated women and minorities may be more successful learning about climate change than white men.

## Introduction

Educational efforts are often promoted as antidotes to apathy and denial associated with environmental issues. Increased environmental knowledge often predicts concerns over the social and environmental impacts of climate change [1], [2], which in turn promotes individual and collective action [3–5]. Despite the effectiveness of education as a tool to promote environmental action, achieving and fostering climate literacy can also be challenging as educational efforts are confounded by successful media campaigns to foster skepticism among the public [6], [7].

Climate change literacy can serve to further polarize certain individuals [8–10]. Kahan et al. (2012) found that scientific literacy and numeracy has opposite effects on climate change concern among those with differing worldviews [11]. They found that those who subscribe to a worldview that ties authority to conspicuous social rankings become less concerned about the risks of climate change with increasing scientific literacy and numeracy. Those with worldviews that favor less regimented forms of social organization and greater collective attention to the individual respond to climate change education with increasing concern. This gap is attributed to identity-protective cognition, wherein the holders of certain worldviews use and credit the information that is supportive of their own values and opinions [8], [11–13]. Adolescents, however, appear more capable of transcending their personal ideology to learn about climate change than adults [14], [15].

Self-perceptions of knowledge represent a less studied factor that may also shape efficacy of climate change education efforts. Perceptions of intelligence can determine how people engage with issues; for example, illusions of superiority can distort the way people filter and take-in information [16], [17]. Studies find that if people feel confident in their levels of knowledge, regardless of their assessed knowledge, they are unlikely to be motivated to acquire new information [18–20]; feelings of academic inferiority, on the other hand, can drive the search for further knowledge [18], [20], [21]. Alternatively, negative self-perceptions can have detrimental impacts; studies find low confidence is one contributing factor to the high attrition rates of women and minorities in science, technology, engineering, and math (STEM) fields [22], [23].

Negative cultural stereotypes can lead to these biased self-perceptions by inducing fear and anxiety—known as stereotype threat [24]. Stereotype threat undermines academic performance and can reduce one’s sense of agency [23], [25]. Negative stereotypes exist in particular for women and minority groups. Both men and women perceive women to be less knowledgeable on the topics of politics [26] and science [27], and studies find individuals with lighter skin are perceived to be more intelligent, regardless of their assessed intelligence [28].

Few studies have explored the knowledge perceptions of women and minorities as it relates to climate change science. This is a critical gap within the climate change perceptions literature for several reasons. First, although women appear to be more ideologically receptive to climate change education than men [7], [29], they may possess self-perceived limitations, intensified by educational attainment [30]-[32], that could influence the way they engage with this increasingly important topic [33], [34]. In addition, the demographics of America are shifting; the US Census Bureau (2012a) predicts the US will be a majority-minority country as early as 2043 [35]. This means that non-whites will be increasingly exposed to, and required to respond to, the impacts of climate change in America. Ensuring that these groups feel empowered to take on such a challenge is therefore important.

Our goal was to evaluate the self-perceptions of knowledge on climate change among various socio-demographic groups, particularly women and minorities. We tested whether the following: age, education, gender, experience with disaster and drought, race, and an interaction between gender and education, predicted the respondent’s self-perceived level of climate change knowledge. Because women may underestimate their comprehension of climate science [32], we anticipated that women would perceive a lower level of climate change knowledge than their male counterparts. We also expected that increasing educational attainment would further reduce the level of knowledge perceived by women, since increasing education may reduce confidence in knowledge among women [30], [31]. Similarly, we hypothesized that minority respondents would self-report less knowledge on climate change than white respondents because previous research suggests racial minorities experience negative self-perceptions of intelligence [23].

## Methods

### Study site

We conducted our surveys in Raleigh, NC, which is a city comprised of a diverse and educated community, built around professional and technical services (Location Quotient = 1.78) and education services (Location Quotient = 0.98) [36], [37]. The percentage of the population with a bachelor’s degree or higher in Raleigh (49.2%), is greater than the average for the state of North Carolina (29%) [38]. Raleigh’s population (pop. 464,758) is also relatively diverse compared to the state as a whole; 40.4% of the population in Raleigh is a minority race or ethnicity, compared to 29.2% statewide [38]. Thus findings from this study are most relevant to other urban areas with an emerging economy and culture linked to technology, professional services, and educational services.

### Survey instrument

We measured respondent’s self-perceived knowledge of climate change on a 5-point scale with the statement “I feel knowledgeable about climate change.” Our survey instrument also included demographic questions about race, age, income, gender, and education, as well as respondent’s experience with natural disasters and drought. We measured education on an 11-point scale, ranging from no formal education completed (1) to a doctorate degree (11). Income was measured in $20,000 increments, ranging from less than $10,000 a year (below poverty) to more than $210,000 a year. The design of the survey instrument was guided by Dillman’s tailored design method [39]. The final instrument was developed with pretesting among an equal number of respondents across five social vulnerability classes (n = 20). Respondents were asked at the end of the survey to give feedback on the instrument. After evaluating this feedback, cognitive interviews were conducted with 5 participants to identify any alternative interpretations of survey questions. All research was reviewed and approved by the NC State University Institutional Review Board for the Use of Human Subjects in Research (Protocol Number 4087) and respondents before each survey provided written informed consent.

### Sampling

We administered two hundred surveys door-to-door in Raleigh, NC, during the summer of 2015. To promote a representative sample of demographic groups, the sample locations were evenly stratified across five social vulnerability classes, ranging from high to low vulnerability, found in Cutter et al.’s 2006–10 [40] social vulnerability index (SoVI) data set. The 2006–10 SoVI data set is a national index of social vulnerability, comprising 27 sociological characteristics that are aggregated into quintiles. Eight significant components, including race and gender, explained 78% of the variance in the SoVI data set [40]. Within the boundary of Raleigh’s city limits, we randomly selected forty households from each of the five SoVI levels using Hawth’s tools in ArcMap 10.x GIS software [41]. We started sampling from those houses selected with the GIS analysis and, if no one answered, we visited every other house within the SoVI boundaries until a participant agreed to be surveyed.

### Data analysis

We constructed a full ordinary least squares regression model in SAS version 9.4 software for Windows [42], for the dependent variable, “I feel knowledgeable about climate change.” We controlled for the following predictor variables: age, race, education, gender, past experience with any natural disasters and drought, and included an interaction term between education and gender. We collapsed the seven race categories into white and minority due to low response rates in several categories. Income was not included in the full model as it was collinear with educational attainment (*r =* 0.45).

## Results

**Fig 1:**
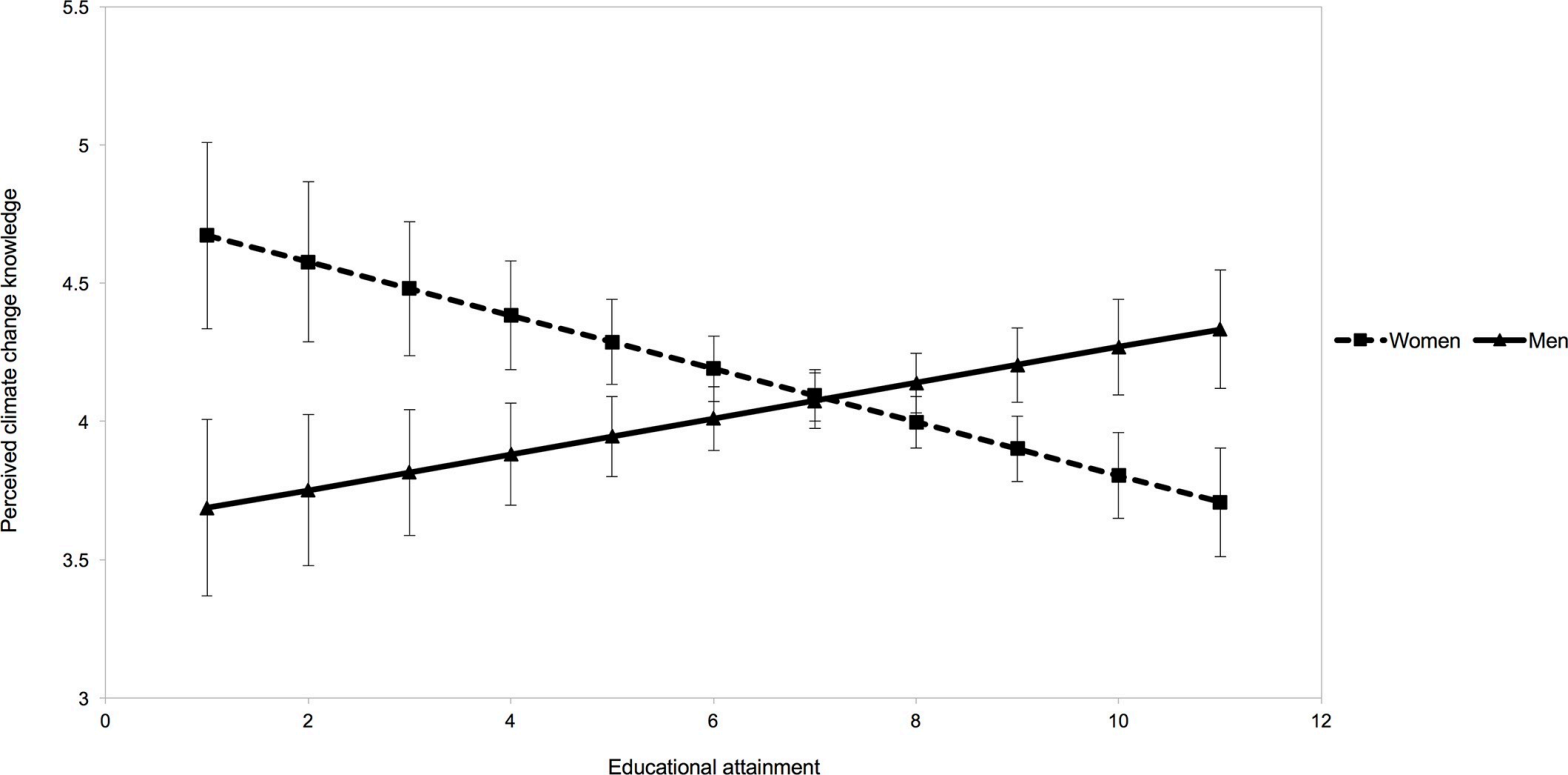
Effect of education on self-perceived climate change knowledge among women and men. Both regression lines predicting climate change knowledge were generated from the regression equation used to generate results in Table 1—where demographic variables, as well as drought and disaster experience predict climate change knowledge. Error bars represent standard error. Y-axis starts at 3 as no respondents self-reported knowledge levels below 3.

Overall, self-perception of climate knowledge was higher among women than men, countering our initial hypothesis (Table 1). However, education interacted with gender: highly educated women had a lower self-perception of climate change knowledge than less educated women, whereas highly educated men had a higher self-perception of climate change knowledge than less educated men (Table 1; Fig 1). Increasing educational attainment reversed the gap between the genders because self-perceived knowledge levels among women declined as their educational attainment increased. With all other variables held constant, women were around one point higher than men on the self-reported climate change knowledge scale at the lowest level of educational attainment, and around 0.6 points lower than men at the highest level of educational attainment. Minority respondents also perceived lower levels of knowledge about climate change than white respondents (*p =* 0.02).

**Table 1:** Predictors of perceived climate change knowledge.

Variable	Beta	Standard error	*p* value
Intercept	3.20	0.46	<0.0001
Age	0.01	0.00	0.14
Education	0.06	0.05	0.19
Gender	1.15	0.51	0.03*
Drought	0.09	0.05	0.12
Disaster	0.03	0.04	0.41
Education x gender	-0.16	0.07	0.02*
Race	-0.35	0.15	0.02*

Education was coded on an 11-point scale from (1) no formal education completed to (11) doctorate degree. Gender was coded as 0 = male and 1 = female. Race was coded as 0 = white and 1 = minority. Drought and disaster were on a 5-point Likert scale ranging from (1) not at all affected to (5) very affected. Model Fit statistics: R^2^ = 0.089, Adj R^2^ = 0.055, *p =* 0.01*, RMSE = 0.92

## Discussion

Our results contribute to literature on self-perceived climate knowledge by suggesting gender differences may be contingent on educational attainment. Women have been shown to underestimate their competence and intelligence in many fields, including climate change [32], [33], a phenomenon commonly referred to as the “gender confidence gap” [43], [44]. The confidence gap is often attributed to the high standards of perfection to which women hold themselves [45], [46], as well as their risk-aversion [47], [48]. However, our results suggest this gap exists primarily among those with higher education and is perhaps reversed between the genders among those with low education.

Multiple explanations for this interaction are possible. Negative stereotypes face women regarding their academic proficiency, especially in science, technology, engineering, and math (STEM) fields [24], [49]. These negative stereotypes can elicit a disruptive and anxious state, known as stereotype-threat [24], [50], [51], which can undermine academic performance and contribute to the high attrition rates of women in STEM fields [22], [23]. These stereotypes can also influence the way women and young girls are treated in classrooms; some teachers are biased against women, particularly in math and the sciences [52], [53], a phenomenon commonly referred to as expectation bias [54], [55]. These biases can further exacerbate the negative self-perceptions of female students [56], [57], and become self-fulfilling prophecies, as these students are often sent on less ambitious tracks [58].

The negative self-perceptions of knowledge held by educated women may actually be an indication of intelligence. Kruger and Dunning [21] illustrated that the highest performing individuals underestimate their abilities, while those who over estimate, but are incompetent, do not recognize their incompetence because they lack metacognition. The “Dunning-Kruger” effect, as it is known, is supported by the climate change literature, where women exhibit more scientifically accurate climate change knowledge than do men, yet underestimate their climate change knowledge more than men [32], [33]. To better explain the differences in perceived knowledge between the genders observed in this and other studies, future studies should assess the actual and perceived levels of knowledge of climate change science between males and females throughout their educational careers.

One crucial implication of the “Dunning-Kruger effect” is that a low level of confidence in one’s own knowledge may actually be a driving force in the search for further information [18], [20]. Highly educated women may therefore be well suited to learning about the complex and controversial topic of climate change. Climate change can be an intrinsically challenging phenomenon to understand [9] because political ideology promotes selective use of information [59] and the causes are not directly observable [60], [61]. The critical thinking skills provided by higher education [62], a healthy skepticism of ones own assumptions, and a drive to seek further knowledge—qualities that highly educated women likely possess, may aid in promoting a better understanding of climate science.

Cultural exclusion, expectation bias, and limited representation of minorities in STEM majors [63] likely all contributed to the low level of self-reported climate change knowledge among minority respondents. Minority groups are often culturally excluded from rigorous scientific and environmental education [64] and many attend schools in low-income urban school districts, which are largely under-funded [65]. Additionally, minority respondents may have self-reported lower levels of knowledge due to cultural stereotypes of intellectual ability, similar to those that plague women. For example, Steele and Aronson [24] found that black students only underperform compared to white students when they believe their intellectual ability is being tested. The biased expectations that teachers have of minority students can influence their pedagogical effectiveness, further driving the academic underperformance of minorities [66]. Future studies should explore the drivers of low perceived climate change knowledge among minorities and compare the levels of assessed climate change knowledge of minority groups to their levels of perceived knowledge.

Increasing climate change knowledge and self-perceptions of intelligence of minority groups is critical since we are projected to be a majority-minority country by 2043 [35]. The lack of psycho-social support coupled with lower perceptions of knowledge could have negative implications for the engagement of racial minorities and women in climate change advocacy and education. More culturally diverse communicators on the topic of climate science could help encourage engagement [11], [67] as well as better k-12 environmental education programs in majority-minority schools. Studies have also found that one way to successfully fortify students against these ill effects is with social-psychological interventions, such as with praise to enforce an incremental theory of intelligence [68] or through self-affirming writing assignments that aim to reduce negative stereotypes [69], [70].

## Conclusion

This study enhances our understanding of the gender gap previously identified in the climate change knowledge perceptions literature [32], [33]. Our results illustrate that the negative perceptions of climate change knowledge in women are correlated with higher educational attainment, and at lower levels of education, women actually self-report more knowledge than men. Although further research is required, previous studies find minorities have lower levels of assessed climate change knowledge [32], and a lack of psycho-social support—which can lead to less engagement with scientific topics [67], [71]. Together with our findings, these studies may indicate the need for educational and psychological intervention on behalf of minorities in order to increase climate change literacy and perceptions of intelligence. A lack of confidence in one’s own knowledge can make educational efforts more effective by encouraging further knowledge seeking [18], but a downside may emerge if highly educated women and minorities avoid starting conversations with others about climate change because they do not believe they understand the issue well enough. Encouraging knowledge-seeking behavior among women and minorities–who are less skeptical about climate change than white men [29]–could help to increase concern and policy action in the politically polarized United States.

## Supporting information

S1 File Educational attainment dataset. Household survey data collected in the summer of 2015.
(XLSX)
